# High Frequency of Pulmonary Hypertension-Causing Gene Mutation in Chinese Patients with Chronic Thromboembolic Pulmonary Hypertension

**DOI:** 10.1371/journal.pone.0147396

**Published:** 2016-01-28

**Authors:** Qunying Xi, Zhihong Liu, Zhihui Zhao, Qin Luo, Zhiwei Huang

**Affiliations:** 1 State Key Laboratory of Cardiovascular Disease, Fuwai Hospital, National Center for Cardiovascular Diseases, Chinese Academy of Medical Sciences and Peking Union Medical College, Beijing, China; 2 Center for Pulmonary Vascular Diseases, State Key Laboratory of Cardiovascular Disease, Fuwai Hospital, National Center for Cardiovascular Diseases, Chinese Academy of Medical Sciences and Peking Union Medical College, Beijing, China; University of Illinois College of Medicine, UNITED STATES

## Abstract

The pathogenesis of chronic thromboembolic pulmonary hypertension (CTEPH) is unknown. Histopathologic studies revealed that pulmonary vasculature lesions similar to idiopathic pulmonary arterial hypertension (PAH) existed in CTEPH patients as well. It’s well-known that genetic predisposition plays an important role in the mechanism of PAH. So we hypothesized that PAH-causing gene mutation might exist in some CTEPH patients and act as a background to facilitate the development of CTEPH. In this study, we analyzed 7 PAH-causing genes including *BMPR2*, *ACVRL1*, *ENG*, *SMAD9*, *CAV1*, *KCNK3*, and *CBLN2* in 49 CTEPH patients and 17 patients recovered from pulmonary embolism (PE) but without pulmonary hypertension(PH). The results showed that the nonsynonymous mutation rate in CTEPH patients is significantly higher than that in PE without PH patients (25 out of 49 (51%) CTEPH patients *vs*. 3 out of 17 PE without PH patients (18%); p = 0.022). Four CTEPH patients had the same point mutation in *ACVRL1* exon 10 (c.1450C>G), a mutation approved to be associated with PH in a previous study. In addition, we identified two CTEPH associated SNPs (rs3739817 and rs55805125). Our results suggest that PAH-causing gene mutation might play an important role in the development of CTEPH.

## Introduction

Chronic thromboembolic pulmonary hypertension (CTEPH) constitutes Group 4 of the Dana Point classification of pulmonary hypertension (PH) and is considered an uncommon sequel of acute pulmonary thromboembolism (PE). The cumulative incidences of CTEPH range between 1.5% and 5.1% of survived PE [1,2,3]. Why these patients who have survived an acute PE continue to develop CTEPH is largely unknown.

The characteristics of CTEPH is the presence of unresolved thromboemboli undergoing fibrotic organisation. Although CTEPH is understood as a thromboembolic disorder, neither classical plasmatic risk factors for venous thromboembolism nor defects in fibrinolysis are found to be associated with CTEPH [4]. And secondary remodeling processes are believed to occur in distal pulmonary vascular bed of CTEPH patients, which is histologically indistinguishable from idiopathic pulmonary arterial hypertension (PAH) [5]. The pathophysiological mechanism of PAH is, increasingly clear, that contributing factors (such as hypoxia, drugs, toxins, inflammation, etc.) involving the action of vasoconstrictive and remodeling processes on a background of genetic predisposition [6]. Genetic predisposition caused by heterogeneous germline mutation of the bone morphogenetic protein type Ⅱ receptor (BMPR2) gene (*BMPR2*) has been found to account for approximately 75% of patients with heritable PAH, and up to 25% of idiopathic PAH [7]. Other genes which have been reported to be involved in the PAH pathogenesis so far are: partners in the transforming growth factor (TGF)-β signaling pathway including activin receptor-like kinase type 1 gene (*ACVRL1*) [8], endoglin gene (*ENG*) [9], SMAD9 gene (*SMAD9*) [10], and genes coded for caveolin-1 (*CAV1*) [11], potassium channel subfamily K, member 3 (*KCNK3*) [12], cerebellin 2 (*CBLN2*) [13]. The inheritance pattern of PAH, which is highly variable and with incomplete expression, implies the existence of environmental modifiers with the capability of modulating disease susceptibility. Since CTEPH share similar histopathological vascular lesions to idiopathic PAH and hereditary PAH, we hypothesized that mutations in heritable or idiopathic PAH genes might be found in patients with CTEPH. And the initial PE event might play as a trigger for facilitating the development of CTEPH.

In the present study, we analyzed the genetic variations in CTEPH patients and patients recovered from PE but without PH to explore whether mutations in known PAH-causing genes played a role in the development of CTEPH.

## Materials and Methods

### Patients

Forty-nine CTEPH patients and 17 PE without PH patients were tested of gene mutation for *BMPR2*, *ACVRL1*, *ENG*, *SMAD9*, *CAV1*, *KCNK3*, and *CBLN2*. All study subjects belonged to the Chinese Han population, and were among patients admitted to the Center for Pulmonary Vascular Diseases of Fuwai Hospital from Oct. 2012 to Mar. 2015. CTEPH was diagnosed as: (1) a mean pulmonary arterial pressure ≥ 25 mmHg with a pulmonary capillary wedge pressure ≤ 15 mmHg based on right heart catheterization; (2) ventilation/perfusion lung scan and/ or computed tomographic pulmonary angiogram, and pulmonary angiography confirmed chronic thromboembolic obstruction. Patients recovered from PE and without PH were defined as (1) normal presentation of echocardiography and computed tomographic pulmonary angiogram during at least 1-year follow-up; (2) undergoing at least 6 months of therapeutic anticoagulation. Detected point mutations were screened in 120 unrelated healthy Chinese individuals to determine polymorphisms. Before participation, all study subjects signed written information consent. The study was approved by the Human Ethics Committee of Fuwai Hospital and conformed to the 1975 Declaration of Helsinki.

### Molecular studies

Genomic DNA was extracted from whole blood with a standard phenol-chloroform protocol. Direct DNA sequencing were performed to detect point mutations and small insertions/ deletions within coding regions and the flanking intron sequences. PCR primers were designed to amplify the coding regions and the intron/exon boundaries of all candidate genes, e.g. *BMPR2*, *ACVRL1*, *ENG*, *SMAD9*, *CAV1*, *KCNK3*, and *CBLN2*. The sequences of each pair of primers and PCR conditions are displayed in Table 1. The coding DNA sequence 1 of *CBLN2* could not amplified by PCR because of technical problem. PCR products were analyzed by using an ABI 3730 XL (Applied Biosystems, Carlsbad, CA, USA). All results were compared with the reference sequences of the known PAH-associated genes including *BMPR2* (GenBank accession no. NM_001204.6), *ACVRL1* (GenBank accession no. NM_000020.2), *ENG* (GenBank accession no. NM_000118.2), *SMAD9* (GenBank accession no. NM_001127217.2), *CAV1* (GenBank accession no. NM_001753.4), *KCNK3* (GenBank accession no. NM_002246.2), and *CBLN2* (GenBank accession no. NM_182511.3). The standard nomenclature recommended by the Human Genome Variation Society (www.hgvs.org/mutnomen) was employed to number mutations. Frequencies of single nucleotide polymorphisms (SNPs) of all candidate genes were compared to those previously reported in the HapMap database (http://hapmap.ncbi.nih.gov/index.html.en).

**Table 1:** The primer sequences used for PCR amplification of coding sequences of 7 pulmonary arterial hypertension-associated genes.

CDS	Forward (5’-3’)Reverse(5’-3’)	Annealing T_m_ (°C)	Product length (bp)
*BMPR2*			
CDS 1	CACCGAAGCGAAACTTAAGG	55	786
	AAGGCGATTTCCCTGGAAG		
CDS 2	GTCATTCGGATAAGACAAA	55	335
	TTTAACATACTCCCATGTCC		
CDS 3	CCCCCCATGAAATGTCTTTG	55	459
	GCCTGGCTTCAACCTTGAAT		
CDS 4	AGGAGCACATCTACTTGGTGTTTT	58	542
	AGTGGCATGGAAAGGGGTAG		
CDS 5	CTCCCAGAATTTGGCTTTCA	55	454
	GTGCCTAGAATAGGCCTTGAC		
CDS 6	CTGGGTCTGGTAGGAGCTTCA	55	532
	CGAGGCTGGTCCTGAACTCT		
CDS 7	CCTTTCCATCCCTTCCTCTC	58	494
	CGTGGGAAAGCTCTTTCTGT		
CDS 8	GAGTTGAAATTCCGATTTCTCTT	58	463
	CCAAGCTGGTCTCGAACTCT		
CDS 9	TCAGGAAGGGCATTTTATAGGT	55	486
	TGCATCCTGCTGCTAATAATGT		
CDS 10	ATGTGCCTGAAGGGGATGAA	58	396
	TTGTGGCATTAGGCAACTCC		
CDS 11	TCCGTAATCCTTGAAGCCTAA	55	525
	GCAGATTTCATCTTGCACTTGT		
CDS 12A	CATTTTTCAGTAGGCTTAATTCAC	55	792
	CTGCTGTCCAGTTGCTTCTAC		
CDS 12B	CAGGACTCACGCCAAGTACTG	55	718
	CGGGTGTCCTCACCAATAAAC		
CDS 12C	GGCAGCAAGCACAAATCAAA	55	684
	CGCCTCAAATGGATCATTTAC		
CDS 13	TGTCTGGCATTATGAATTTCAAG	55	747
	CCTTAAGAAACTGGTCCAAACTG		
*ENG*			
CDS 1	CGGTCATACCACAGCCTTCAT	60	684
	CGTCGCTGCACAGCATTCT		
CDS 2	GCCGTTAGCTCATGTCAAGTC	55	305
	TGCCTTGGAGCTTCCTCTG		
CDS 3	ACAGAGCAGGCAGGGAGAGT	58	336
	AGACCCTGACCCACAGAGATG		
CDS 4	ATTCTCAGCTCCGGCCTCT	60	414
	GAACTGTGGCACAGCGTGTC		
CDS 5	ATCTTTGGCTGTGGGTGAGG	55	401
	GGTGGGGCTTTATAAGGGAC		
CDS 6	ACCTGGCCAGGTAAGAGTGC	58	387
	CACTCCTGCTGCGTCTTCTG		
CDS 7	CGAGCTGAGCTGAAGGACAA	55	428
	ACAGAGGTGCTTCACCAACAGT		
CDS 8	CAGCATTGTGGCATCCTTCG	60	861
	GCCAGCTCAGGGAGCATTTA		
CDS 9	GGGGAATGGCTGTGACTT	55	405
	GGAGACTAAGCCAACCAATG		
CDS 10	CCATTGGTTGGCTTAGTCTC	55	301
	CTGCTCCGGTCATACAGAAG		
CDS 11	AGAGTCAGGCAACTCCACAG	58	384
	CCTGAGCAATGCCTTCTCT		
CDS 12	GACTCAGGGGTGGGAACTC	55	496
	GGCCACATGCCTGATTAAG		
CDS 13	AGCTACGAAGCGGTGGAGAT	55	289
	CCCTTGCCATGTGCTATGTG		
CDS 14A	AGCCCAGTGAAGCCTCTGA	60	466
	TGGCAAGTGGTCTGTCTCCT		
CDS 14B	CAGCCACTGGCTTGGAACA	60	646
	CCTGGATGCTGCTACTGTTCA		
*ACVRL1*			
CDS 1	CAGCGGCTGTCACACTTCAT	58	249
	CACTCTCCAGTCAAGCTCCTACA		
CDS 2	TTCTGAGGGAAGGATGACTGA	58	560
	CAGACCACTCTGCCAGTTAGAT		
CDS 3	GGATCTAACTGGCAGAGTGGT	58	386
	CCACCGGCTCTAATCTCTG		
CDS 4	GAGTGAGGAGCTTGCAGTGAC	55	295
	ACCGCCTGTGATTCCAGTAG		
CDS 5	AGCGCAGCATCAAGATGG	55	309
	AATGTCTGGAGGTCTGCAAACT		
CDS 6	CCACCCCCAGACCTAGCTTA	55	545
	TCTTGCGGAGGAGAACTGAA		
CDS 7	CCAGGTCTGCTCTGTGAAGTG	55	520
	CTGGCTCCACAGGCTGATT		
CDS 8	CTCAGGGGTAGCGTGTCCA	58	344
	AGGCCTCAGACACAAGTTCC		
CDS 9	GGCCATCCTCCTCATCTTCT	58	582
	CTGGCTTGGCACTCTGACTC		
*KCNK3*			
CDS 1	GACGATGAAGCGGCAGAAC	55	433
	GCTGAACTCGGGTTTCTGC		
CDS 2A	TGGACCCAGACCCACAAG	55	575
	TCATGAAGCGCAGCACC		
CDS 2B	CAGTACGTGGCCTTCAGCTT	55	684
	AGCAGGCACAGTCGGAGAT		
*CBLN2*			
CDS 2	GGCAGTGTTGCTGGTTATCC	55	293
	AGCCTCAGAGACCAGGTGAA		
CDS 3	CAAGCTGCCGGTTCTCTATT	55	622
	GAAGGAGGCAGTGCTGAAGTTA		
*SMAD 9*			
CDS 1	TGTGGCCTCTTATGCACTCC	55	614
	TGATTGGACAGCTGCCTCAT		
CDS 2	GTTCCCAAGGGGAAAAACAG	55	559
	TCCAGGGTAACTGCTTCAAAAT		
CDS 3	AGGCTCTCAGAACAACCAGTTT	55	423
	CAAAACAGCAGGCCAGTACA		
CDS 4	GAGCGGCATGCTTGGTCTT	55	515
	TGCTTTCCCCAGTGGTAATG		
CDS 5	TAAGCGGCATTTAGGTCACAC	58	512
	TGTCTATCAGAATTGACCGCACA		
CDS 6	CAGGAAGGTAGAGGCCATGTT	58	731
	CTGTAGGGGTTTCACTGCTTTG		
*CAV1*			
CDS 1	TCCACCCCTGCTGAGATGAT	55	498
	GGCAGCAGTCGGGATATTTG		
CDS 2	GTCGGAGCGGTTAGTTCGAT	55	494
	GTAACGTTTCTGCCGACTGC		
CDS 3	TGTGTTCCCAAGTTCCAAGTG	55	780
	AGCCAATAAAGCGATGGTTGAT		

CDS: coding DNA sequence.

Thirty CTEPH patients and 14 PE without PH patients who no mutation has been detected by direct DNA sequencing undergone multiple ligation probe amplification (MLPA) analysis for *BMPR2*, *ACVRL1*, and *ENG* by using SALSA MLPA1P093 HHT probe mix kit (MRC-Holland, Amsterdam, the Netherlands). Samples were analyzed using an ABI 3730 Genetic Analyzer, and the results were visualized by the Coffalyser software (MRC-Holland).

### Statistical analysis

The demographic and clinical characteristics of different groups were compared using the student’s t tests or the Mann-Whitney U test for continuous variables, and chi-square tests for categorical variables. Differences in the frequencies of targeted gene mutations and the distribution of SNPs between the CTEPH patients and PE without PH patients were evaluated using the Fisher’s exact test. By using the chi-square test, we tested whether the genotype distributions for the studied SNPs were in the Hardy-Weinberg equilibrium. A p value <0.05 were considered as statistical significant. All statistical analyses were performed using the Statistical Package for Social Science (SPSS) 16.0 for Windows (SPSS Inc., Chicago, IL, USA).

## Results

### Clinical characteristics of the study population

The clinical characteristics of the CTEPH patients and PE without PH patients were displayed on Table 2. The mean±SD age was 51±16 years for CTEPH patients, and 53±18 years for PE without PH patients. Forty-nine percent of CTEPH patients and 41% of PE without PH patients were female. The levels of N-terminal pro-brain natriuretic peptide were significantly higher in CTEPH patients than that in PE without PH patients. While levels of oxygen saturation were significantly lower in CTEPH patients compared with the PE without PH patients.

**Table 2:** Clinical characteristics of study subjects.

	CTEPH(n = 49)	PE without PH(n = 17)	p value
Age (years)	51±16	53±18	0.646
Female (%)	24 (49)	7 (41)	0.779
BMI (kg/m^2^)	22.74±3.26	26.13±2.74	<0.001
NT-proBNP (pg/ml)	2408.6±1909.7	315.6±223.5	<0.001
SaO_2_ (%)	91.14±4.43	95.11±3.25	0.001
mPAP (mmHg)	60±14		
PVR (Wood units)	14.16±3.48		
CI (L/min.m^2^)	2.53±0.48		
Previous history of PE	14 (29)		
Distal (%)	14 (29)		

CTEPH: chronic thromboembolic pulmonary hypertension; PE: pulmonary embolism; PH: pulmonary hypertension; BMI: body mass index; NT-proBNP: N-terminal pro-brain natriuretic peptide; SaO_2:_ oxygen saturation; mPAP: mean pulmonary artery pressure; PVR: pulmonary vascular resistance; CI: cardiac index.

### Mutation rate and distribution

Sixty-six (n = 66) sequences were included for analysis. These sequences were obtained from patients with CTEPH (n = 49) and patients recovered from PE and without PH (n = 17). A total of 40 mutations were identified, including 31 point mutations and 9 large size rearrangements (Table 3). Among them, 5 mutations were synonymous (3 in CTEPH patients, 2 in PE without PH patients).

**Table 3:** categories of gene mutations in CTEPH patients and PE without PH patients.

Patient	Point mutation			large size rearrangement	Total
	Synonymous	missense	VUS		
CTEPH	3	16	9	5	33
PE without PH	2	1	0	4	7
Total	5	17	9	9	40

CTEPH: chronic thromboembolic pulmonary hypertension; PE: pulmonary embolism; PAH: pulmonary arterial hypertension; VUS: variant of unknown significance.

Point mutation analysis is summarized in Table 4. Portions of these transitions were subsequently analyzed in 120 unrelated healthy Chinese individuals (Table A in S1 File). PolyPhen-2 was employed to predict the effect on protein function. Thirteen mutations were predicted to be probably damaging, 2 mutation was predicted to be possibly damaging, and 2 mutations were predicted to be benign. Three patients had multiple point mutations.

**Table 4:** Gene mutations in Chinese CTEPH patients and PE without PH patients.

Patientsnumber	Group	Gene	Location	Nucleotide change	Amino acid change	Mutation type
54	CTEPH	*BMPR2*	5’-UTR	c.-93A>G	p.?	VUS
183	PE without PH		Exon3	c.292G>A	Glu98Lys	Missense
101	CTEPH		Exon11	c.1569T>C	Thr523Thr	Synonymous
281	CTEPH		Exon12	c.1739G>T*	Gly580Val	Missense
198	CTEPH		Exon12	c.2030T>A*	Let677His	Missense
275	CTEPH		Exon12	c.2006A>G*	Asp669Gly	Missense
285	CTEPH		Exon12	c.2325C>A	Ser775Arg	Missense
248	CTEPH		Exon12	c.2357C>G*	Thr786Ser	Missense
121	CTEPH		Exon12	c.2663T>G	Val888Gly	Missense
198	CTEPH	*ENG*	Intron13	c.111+62A>G	p.?	VUS
77	CTEPH		3’-UTR	c.124+277A>G	p.?	VUS
248	CTEPH	*ACVRL1*	Exon5	c.583C>T*	Gln195Trp	Missense
198	CTEPH		Exon8	c.1196G>T*	Trp399Leu	Missense
2	CTEPH		Exon10	c.1450C>G*	Arg484Gly	Missense
191	CTEPH		Exon10	c.1450C>G*	Arg484Gly	Missense
263	CTEPH		Exon10	c.1450C>G*	Arg484Gly	Missense
285	CTEPH		Exon10	c.1450C>G*	Arg484Gly	Missense
274	CTEPH	*KCNK3*	Exon1	c.92C>A*	Ser31Trp	Missense
117	PE without PH		Exon2	c.414C>T	Tyr138Tyr	Synonymous
262	CTEPH	*CBLN2*	3’-UTR	c.200+108G>C	?	VUS
285	CTEPH		3’-UTR	c.200+630T>C	?	VUS
211	PE without PH		Exon5	c.648T>C	Phe216Phe	Synonymous
285	CTEPH	*SMAD9*	5’-UTR	c.-98C>T	?	VUS
278	CTEPH		Exon2	c.260T>A*	Leu87Gln	Missense
285	CTEPH		Exon3	c.521A>G*	Asn174Ser	Missense
235	CTEPH	*CAV1*	5’-UTR	c.-106A>G	p.?	VUS
235	CTEPH		5’-UTR	c.-17T>C	p.?	VUS
287	CTEPH		Exon2	c.76A>G	Lys26Glu	Missense
263	CTEPH		Exon3	c.387A>G	Ala129ALa	Synonymous
191	CTEPH		Exon3	c.462C>T	Thr154Thr	Synonymous
124	CTEPH		3’-UTR	c.341+274A>T	p.?	VUS

CTEPH: chronic thromboembolic pulmonary hypertension; PE: pulmonary embolism; PH: pulmonary hypertension; UTR: untranslated region; VUS: variant of unknown significance. Numbering is based on +1 as A of the ATG initiation codon.

*: missense mutation predicted to be probably damaging by Polyphen-2 software.

The large size rearrangement analysis is summarized in Table 5. Three patients had more than one large size rearrangements.

**Table 5:** Large size rearrangement identified in CTEPH patients and PE without PH patients.

Identification number	Diagnosis	Gene	Rearrangement
270	CTEPH	BMPR2 exon 1	c.1-?_c.76+?del
		ENG exon 6	c.690-?_c.816+?del
		ENG exon 11–14	c.1312-?_c.1977+?del
282	CTEPH	BMPR2 exon 1	c.1-?_c.76+?del
		ENG exon 12	c.1429-?_c.1686+?del
187	PE without PH	ENG exon 14	c.1742-?_c.1977+?dup
603	PE without PH	ENG exon 5	C.524-?_c.689+?dup
		ENG exon 8	c.992-?_1134+?dup
		ENG exon 14	c.1742-?_c.1977+?dup

Abbreviations are in accord with nomenclature guidelines as recommended by the Human Genome Variation Society. CTEPH: chronic thromboembolic pulmonary hypertension; PE: pulmonary embolism; PH: pulmonary hypertension; del: deletion; dup: duplication; c.: coding DNA where nucleotide 1 is the A of the ATG translation initiation codon.

**Fig 1:**
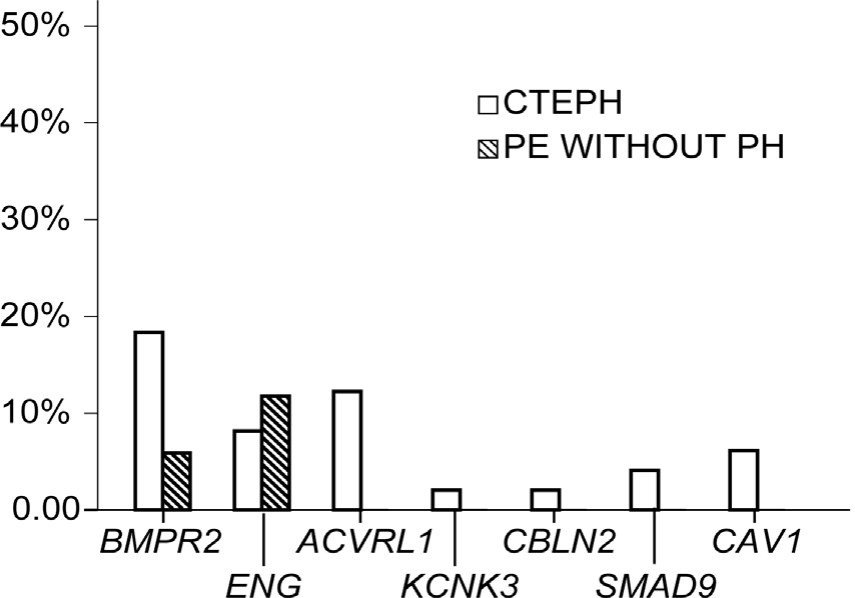
Distribution of nonsynonymous mutations of 7 pulmonary arterial hypertension-causing genes in chronic thromboembolic pulmonary hypertension patients and patients recovered from pulmonary embolism and without pulmonary hypertension.

The nonsynonymous mutation rate in CTEPH patients is significantly higher than that in PE without PH patients (25 out of 49 (51%) CTEPH patients *vs*. 3 out of 17 PE without PH patients (18%); p = 0.022). The mutation rate for individual gene was displayed on Fig 1.

### Polymorphisms

Twelve SNPs which already recorded in the public dbSNP database were detected among all the 66 patients (Table 6). Four single nucleotide changes were considered to be polymorphisms because they were also found in control individuals (Table 6). Genotype distributions of the studied SNPs were all in Hardy-Weinberg equilibrium. We compared the genotype and allele frequencies of these SNPs between the CTEPH patients and the PE without PH patients, and found out that the allele of SNP rs3739817 and SNP rs55805125 were significantly correlated with CTEPH.

**Table 6:** Outline of SNPs.

SNPs	Genotype			P value	Allele		P value	OR
rs140683387	A/A	A/T	T/T		A	T	1.000	-
CTEPH	48(0.98)	1(0.02)	0(0)	1.000	97(0.99)	1(0.01)		
PE without PH	17(1.0)	0(0)	0(0)		34(1.00)	0(0)		
rs369291114	C/C	C/T	T/T		C	T	1.000	-
CTEPH	48(0.98)	1(0.02)	0(0)	1.000	97(0.99)	1(0.01)		
PE without PH	17(1.0)	0(0)	0(0)		34(1.00)	0(0)		
rs1061157	G/G	G/A	A/A		G	A	0.446	2.776(0.36–21.39)
CTEPH	41(0.84)	8(0.16)	0(0)	0.427	90(0.92)	8(0.08)		
PE without PH	16(0.94)	1(0.06)	0(0)		33(0.97)	1(0.03)		
rs148475405	C/C	C/T	T/T		C	T	0.450	0.347(0.02–5.40)
CTEPH	48(0.98)	1(0.02)	0(0)	0.452	97(0.99)	1(0.01)		
PE without PH	16(0.94)	1(0.06)	0(0)		33(0.97)	1(0.03)		
rs11545664	G/G	G/A	A/A		G	A	1.000	1.041(0.11–9.67)
CTEPHPE without PH	46(0.94)16(0.94)	3(0.06)1(0.06)	0(0)0(0)	1.000	95(0.97)33(0.97)	3(0.03)1(0.03)		
rs1800956	G/G	G/C	C/C		G	C	1.000	0.925(0.26–3.29)
CTEPH	43(0.88)	4(0.08)	2(0.04)	0.404	90(0.92)	8(0.08)		
PE without PH	14(0.82)	3(0.18)	0(0)		31(0.91)	3(0.09)		
rs3739817	C/C	C/T	T/T		C	T	**0.038**	0.173(0.03–0.91)
CTEPH	48(0.98)	0(0)	1(0.02)	**0.003**	96(0.98)	2(0.02)		
PE without PH	13(0.76)	4(0.24)	0(0)		30(0.88)	4(0.12)		
rs55805125	C/C	C/T	T/T		C	T	**0.016**	0.087(0.01–0.75)
CTEPH	48(0.98)	1(0.02)	0(0)	**0.014**	97(0.99)	1(0.01)		
PE without PH	13(0.76)	4(0.24)	0(0)		30(0.88)	4(0.12)		
rs199874575	C/C	C/T	T/T		C	T	0.300	-
CTEPH	49(1.00)	0(0)	0(0)	0.258	98(1.00)	0(0)		
PE without PH	16(0.94)	1(0.06)	0(0)		33(0.98)	1(0.02)		
rs368057934	C/C	C/T	T/T		C	T	1.000	-
CTEPH	48(0.98)	1(0.02)	0(0)	1.000	97(0.99)	1(0.01)		
PE without PH	17(1.00)	0(0)	0(0)		34(1.00)	0(0)		
rs549923058CTEPH	G/G48(0.98)	G/A1(0.02)	A/A0(0)	1.000	G97(0.99)	A1(0.01)		
PE without PH	17(1.00)	0(0)	0(0)		34(1.00)	0(0)	1.000	-
rs79733377	C/C	C/A	A/A		C	A	0.450	0.347(0.02–5.40)
CTEPH	48(0.98)	1(0.02)	0(0)	0.452	97(0.99)	1(0.01)		
PE without PH	16(0.94)	1(0.06)	0(0)		33(0.97)	1(0.03)		
*ENG* c.-59C>A	C/C	C/A	A/A		C	A	1.000	-
CTEPH	48(0.98)	1(0.02)	0(0)	1.000	97(0.99)	1(0.01)		
PE without PH	17(1.00)	0(0)	0(0)		34(1.00)	0(0)		
CBLN2 exon5								
c.527A>C	C/C	C/A	A/A		C	A	0.230	1.388(0.84–2.28)
CTEPH	12(0.24)	26(0.53)	11(0.23)	0.365	50(0.51)	48(0.49)		
PE without PH	7(0.41)	8(0.47)	2(0.12)		22(0.65)	12(0.35)		
CBLN2 exon5								
c.628T>C	T/T	T/C	C/C		T	C	1.000	1.388(0.31–6.22)
CTEPH	2(0.04)	4(0.08)	43(0.88)	0.704	8(0.08)	90(0.92)		
PE without PH	0(0)	2(0.12)	15(0.88)		2(0.06)	32(0.94)		
SMAD9 exon3								
c.487G>C	G/G	G/C	C/C		G	C	1.000	-
CTEPH	48(0.98)	1(0.02)	0(0)	1.000	97(0.08)	1(0.92)		
PE without PH	17(0)	0(0)	0(0)		34(1.00)	0(0)		

SNP: single nucleotide polymorphism; CTEPH: chronic thromboembolic pulmonary hypertension; PE: pulmonary embolism; PAH: pulmonary arterial hypertension.

### Clinical characteristics of CTEPH patients in mutation carriers and noncarriers

There is no significance of gender, age, previous PE history, and hemodynamic parameters between the mutation carriers and noncarriers CTEPH patients. However, BMI in mutation carriers are lower than the noncarriers (Table 7). Detailed information for each patient was displayed on Table B in S1 File.

**Table 7:** Comparison of clinical characteristics of CTEPH patients in nonsynonymous mutation carriers and noncarriers.

Variable	Mutation carrier(n = 19)	Noncarrier(n = 30)	p value
Age (years)	51±15	51±17	0.918
Female (%)	9 (53)	14 (47)	0.684
BMI (kg/m^2^)	21.55±2.53	23.78±3.56	0.022
NT-proBNP (pg/ml)	2311.0±2245.8	2474.9±1685.2	0.776
SaO_2_ (%)	92.23±3.44	90.53±4.80	0.188
mPAP (mmHg)	59±16	61±12	0.640
PVR (Wood units)	13.29±2.64	14.09±3.04	0.345
CI (L/min.m^2^)	2.68±0.43	2.52±0.44	0.239
Previous history of PE (%)	5 (26)	9 (30)	0.781
Distal (%)	5 (26)	8 (27)	0.978

CTEPH: chronic thromboembolic pulmonary hypertension; BMI: body mass index; NT-proBNP: N-terminal pro-brain natriuretic peptide; mPAP: mean pulmonary artery pressure; PVR: pulmonary vascular resistance; CI: cardiac index; PE: pulmonary thromboembolism.

## Discussion

The present study is the first to investigate the association between the genotype of PAH-causing genes and the development of CTEPH. We screened 7 known PAH-causing genes in 49 Chinese CTEPH patients and 17 PE without PH patients. We found that CTEPH patients had a higher frequency of mutations in PAH-causing genes compared with PE without PH patients. And we also found two previously identified SNPs (rs3739817 and rs55805125) were significantly correlated with CTEPH.

The underlying mechanism of CTEPH is largely unknown. Since the characterization of CTEPH is the presence of unresolved thromboemboli undergoing fibrotic organization and secondary remodeling processes of the pulmonary vascular bed [4], previous researches have focused on the imbalance between thrombosis and fibrinolysis. However, no correlation has been found between CTEPH and the classical thromboembolic risk factors such as the deficiency of antithrombin, protein C and protein S, the gene mutation of prothrombin, factor V Leiden, and hyperhomocysteinaemia [14]. In one research, the investigators found that fibrin derived from CTEPH patients was resistant to lysis compared with the healthy controls [15]. But a recent study showed that fibrin resistance to lysis occurred in PAH other than CTEPH and, to a smaller extent, in prior PE without PH patients [16]. These findings imply that CTEPH might develop in a more insidious way other than abnormal coagulation and fibrinolysis, especially in those patients who develop CTEPH after the first episode of PE. This assumption seems plausible if we take it into consideration that only a small part of PE patients developed CTEPH, though the percentage of patients with residual pulmonary thrombi was as high as 52% after 11 months of PE diagnosis [17]. In a series of histopathologic studies in CTEPH patients, researchers reported that there were not only the thrombi obstructions in large vessels, but lesions similar to PH including plexogenic lesions were observed under microscopy [18,19,20]. And these typical plexogenic lesions distributed in both of the no-flow lung tissues and normal-flow lung tissues [20]. These findings suggest that CTEPH might develop in a mechanism similar to PAH.

It’s acknowledged that gene mutation play a pivotal role in PAH. The inheritance pattern is complex due to the incomplete penetrance. The penetrance is influenced by environmental factors such as hormone, appetite-suppressant drugs, concurrent inflammation, reactive oxygen species formation [7]. So we hypothesized that genetic predisposition for PAH might exist in some CTEPH patients, and the PE event might act as a second genetic hit within the pulmonary vasculature to promote the mutation gene expression, which lead to endothelial cell proliferation and finally development of CTEPH. *BMPR2* was identified as the first PAH-causing gene in 2000 by two teams of investigators. After that, *AVCRL1* (or *ALK1*) and *ENG* were reported as the PAH-causing genes in 2001 and 2004 respectively [8,9]. These three genes are all belong to the transforming growth factor-beta (TGF-β) receptor superfamily. The TGF-β superfamily comprises a large series of cytokine growth factors that controls a host of cellular functions, including proliferation, migration, differentiation, apoptosis, and extracellular matrix secretion and deposition. In 2009, several teams identified mutation in *SMAD9*, another member of TGF-β superfamily, in patients with PAH [7]. In 2012, a mutation in *CAV1* (codes for caveolin-1, a membrane protein of caveolae abundant in the endothelium and other cells of the lung) was identified in hereditary PAH patients [11]. In 2013, two novel genes, *KCNK3* (the gene encoding potassium channel subfamily K, member 3) and *CBLN2* (codes for cerebellin 2) were detected in hereditary and idiopathic PAH patients [12,13].

In the present study, we identified 25 nonsynonymous point mutations and 5 large size rearrangements in 19 out of 49 CTEPH patients, including 9 mutations in *BMPR2*, 5 mutations in *ENG*, 6 mutations in *ACVRL1*, 1 mutation in *KCNK3*, 2 mutations in *CBLN2*, 3 mutations in *SMAD9*, and 4 mutations in *CAV1*. While in 17 PE without PH patients, 1 patient with *BMPR2* point mutation and 2 patients with 4 large size rearrangements of *ENG* were identified. *BMPR2* is the most studied PAH-causing gene. It comprises 13 exons and harbors four distinct functional domains. Over 300 *BMPR2* unique mutations have been reported so far [21], which distribute in all 13 exons. Six missense mutation of *BMPR2* identified in CTEPH patients in the present study were confined to exon 12, which codes for the cytoplasmic tail. Four of these 6 missense mutations were predicted to be probably damaging by Polyphen-2 software. Surprisingly, we found one mutation in exon 3 in PE without PH patients. Exon 3 codes for an extracellular binding ligand. But the mutation was predicted to be possible damaging by Polyphen-2 software with a score of 0.515. A thorough history and clinical examination did not reveal any sign for PH in this patient or his family. All 6 missense mutations in *ACVRL1* detected in CTEPH were predicted to be probably damaging with scores of 1.0. The mutations were located in exon 5, 8, 10. *ACVRL1* exon 5 codes for the glycine- serine domain, and exons 6–10 code for the kinase domain, all are critical for signaling activity of *ACVRL1*. Four CTEPH patients had the same point mutation in *ACVRL1* exon 10 (c.1450C>G), a variant which had been observed cosegregated with PH in a hereditary hemorrhagic telangiectasia kindred [8]. And histologic assessment had demonstrated that the affected patients had characteristic features of end-stage plexogenic PH similar to those seen in patients with idopathic PH.

The SMAD signal pathway is most strongly associated with driving the differentiation state in development. And the *SMAD9* variants are more convincingly associated with PAH among all *SMADs* according to previous researches [7]. In the present study, we found 2 *SMAD9* missense mutations, which were predicted to be probably damaging. *KCNK3* encodes an pH-sensitive potassium channel protein that contains two pore-forming P domains. *KCNK3* channels are major contributors to the resting potential in human pulmonary-artery smooth muscle cells. Gurney AM, et al [22] suggested that the pH-sensitive channels could be responsible for the modulatory effects of pH on hypoxic pulmonary vasoconstriction. In the present study, we found a missense mutation in *KCNK3* exon 1 in one CTEPH patient. The mutation was predicted to be probably damaging with a score of 1.0. It is plausible that the *KCNK3* mutation might play an important role in CTEPH after the initial PE-induced hypoxia in pulmonary vasculature. *CAV1* encodes caveolin-1, which is the predominant member of three proteins (caveolin-1, caveolin-2, and caveolin-3) that coat the flask-like invaginations of the plasma membrane known as caveolae. Signaling cascades relevant to PAH such as the TGF-β superfamily, nitric oxide pathway, and G-protein coupled receptors rely heavily on proper caveolar function [23]. We identified one missense mutation in *CAV1* exon2, which was predicted to be possible damaging.

In addition, we found mutations in untranslated regions and introns of *BMPR2*, *ENG*, *SMAD9*, *CAV1*, and *CBLN2* (encodes cerebellin 2, which highly expressed in lungs of PAH patients). And we found a significant association between the genotypes of 2 SNPs rs3739817 (*ENG* exon8) and rs55805125 (*ACVRL1* exon 7) with CTEPH. Although there was no encoding amino acid changes resulting from these mutations and polymorphisms, they may modify gene expression, or cause aberrant pre-mRNA splicing due to alternations in sequences recognized by various splicing factors.

Lack of functional analyses of these mutations is the main limitation of the present study. But previous study provided a convincing evidence for the association between one specific mutation in *ACVRL1* exon 10 with PH. Besides, the relatively small sample of patients hindered a thorough mapping of PAH-causing genes variants in CTEPH patients.

## Conclusions

Based on direct sequencing and MLPA method, we identified 25 nonsynonymous point mutations, 5 large size rearrangements, and 2 CTEPH associated SNPs of 7 PAH-causing genes (*BMPR2*, *ACVRL1*, *ENG*, *SMAD9*, *CAV1*, *KCNK3*, *and CBLN2*) out of 49 CTEPH patients. Among them, one specific mutation in *ACVRL1* exon 10 shared by 4 CTEPH patients had been approved to be associated with PH in a previous study. The high frequency of mutations of PAH-causing genes might be associated with CTEPH in Chinese population.

## Supporting Information

S1 File Gene mutaion in 120 healthy controls (Table A).Clinical characteristics of the CTEPH subjects (Table B). (RAR)

## Abbreviations

BMPR2: bone morphogenetic protein type Ⅱ receptor
CTEPH: chronic thromboembolic pulmonary hypertension
MLPA: multiple ligation probe amplification
PAH: pulmonary arterial hypertension
PE: pulmonary thromboembolism
PH: pulmonary hypertension
SNP: single nucleotide polymorphism
TGF: transforming growth factor
